# A CRISPR/Cas12a-empowered surface plasmon resonance platform for rapid and specific diagnosis of the Omicron variant of SARS-CoV-2

**DOI:** 10.1093/nsr/nwac104

**Published:** 2022-06-03

**Authors:** Zhi Chen, Jingfeng Li, Tianzhong Li, Taojian Fan, Changle Meng, Chaozhou Li, Jianlong Kang, Luxiao Chai, Yabin Hao, Yuxuan Tang, Omar A Al-Hartomy, Swelm Wageh, Abdullah G Al-Sehemi, Zhiguang Luo, Jiangtian Yu, Yonghong Shao, Defa Li, Shuai Feng, William J Liu, Yaqing He, Xiaopeng Ma, Zhongjian Xie, Han Zhang

**Affiliations:** Shenzhen Engineering Laboratory of Phosphorene and Optoelectronics; International Collaborative Laboratory of 2D Materials for Optoelectronics Science and Technology of Ministry of Education; Shenzhen Institute of Translational Medicine; Department of Otolaryngology, Shenzhen Second People's Hospital; the First Affiliated Hospital; Institute of Microscale Optoelectronics, Shenzhen University, Shenzhen 518060, China; Shenzhen Engineering Laboratory of Phosphorene and Optoelectronics; International Collaborative Laboratory of 2D Materials for Optoelectronics Science and Technology of Ministry of Education; Shenzhen Institute of Translational Medicine; Department of Otolaryngology, Shenzhen Second People's Hospital; the First Affiliated Hospital; Institute of Microscale Optoelectronics, Shenzhen University, Shenzhen 518060, China; Shenzhen International Institute for Biomedical Research, Shenzhen 518116, China; Shenzhen Engineering Laboratory of Phosphorene and Optoelectronics; International Collaborative Laboratory of 2D Materials for Optoelectronics Science and Technology of Ministry of Education; Shenzhen Institute of Translational Medicine; Department of Otolaryngology, Shenzhen Second People's Hospital; the First Affiliated Hospital; Institute of Microscale Optoelectronics, Shenzhen University, Shenzhen 518060, China; Shenzhen International Institute for Biomedical Research, Shenzhen 518116, China; Shenzhen Engineering Laboratory of Phosphorene and Optoelectronics; International Collaborative Laboratory of 2D Materials for Optoelectronics Science and Technology of Ministry of Education; Shenzhen Institute of Translational Medicine; Department of Otolaryngology, Shenzhen Second People's Hospital; the First Affiliated Hospital; Institute of Microscale Optoelectronics, Shenzhen University, Shenzhen 518060, China; Shenzhen Engineering Laboratory of Phosphorene and Optoelectronics; International Collaborative Laboratory of 2D Materials for Optoelectronics Science and Technology of Ministry of Education; Shenzhen Institute of Translational Medicine; Department of Otolaryngology, Shenzhen Second People's Hospital; the First Affiliated Hospital; Institute of Microscale Optoelectronics, Shenzhen University, Shenzhen 518060, China; Shenzhen Engineering Laboratory of Phosphorene and Optoelectronics; International Collaborative Laboratory of 2D Materials for Optoelectronics Science and Technology of Ministry of Education; Shenzhen Institute of Translational Medicine; Department of Otolaryngology, Shenzhen Second People's Hospital; the First Affiliated Hospital; Institute of Microscale Optoelectronics, Shenzhen University, Shenzhen 518060, China; Shenzhen Engineering Laboratory of Phosphorene and Optoelectronics; International Collaborative Laboratory of 2D Materials for Optoelectronics Science and Technology of Ministry of Education; Shenzhen Institute of Translational Medicine; Department of Otolaryngology, Shenzhen Second People's Hospital; the First Affiliated Hospital; Institute of Microscale Optoelectronics, Shenzhen University, Shenzhen 518060, China; Shenzhen Engineering Laboratory of Phosphorene and Optoelectronics; International Collaborative Laboratory of 2D Materials for Optoelectronics Science and Technology of Ministry of Education; Shenzhen Institute of Translational Medicine; Department of Otolaryngology, Shenzhen Second People's Hospital; the First Affiliated Hospital; Institute of Microscale Optoelectronics, Shenzhen University, Shenzhen 518060, China; Shenzhen Engineering Laboratory of Phosphorene and Optoelectronics; International Collaborative Laboratory of 2D Materials for Optoelectronics Science and Technology of Ministry of Education; Shenzhen Institute of Translational Medicine; Department of Otolaryngology, Shenzhen Second People's Hospital; the First Affiliated Hospital; Institute of Microscale Optoelectronics, Shenzhen University, Shenzhen 518060, China; Shenzhen Han's Tech Limited Company, Shenzhen 518000, China; Shenzhen Engineering Laboratory of Phosphorene and Optoelectronics; International Collaborative Laboratory of 2D Materials for Optoelectronics Science and Technology of Ministry of Education; Shenzhen Institute of Translational Medicine; Department of Otolaryngology, Shenzhen Second People's Hospital; the First Affiliated Hospital; Institute of Microscale Optoelectronics, Shenzhen University, Shenzhen 518060, China; Shenzhen Metasensing Tech Limited Company, Shenzhen 518000, China; Department of Physics, Faculty of Science, King Abdulaziz University, Jeddah 21589, Saudi Arabia; Department of Physics, Faculty of Science, King Abdulaziz University, Jeddah 21589, Saudi Arabia; Research Center for Advanced Materials Science (RCAMS), King Khalid University, Abha 61413, Saudi Arabia; Department of Chemistry, College of Science, King Khalid University, Abha 61413, Saudi Arabia; Zhongmin (Shenzhen) Intelligent Ecology Co. Ltd, Shenzhen 518055, China; Shenzhen International Institute for Biomedical Research, Shenzhen 518116, China; Key Laboratory of Optoelectronic Devices and Systems of Ministry of Education and Guangdong Province, College of Physics and Optoelectronic Engineering, Shenzhen University, Shenzhen 518060, China; Department of Laboratory Medicine, Shenzhen Children's Hospital, Shenzhen 518038, China; Optoelectronics Research Center, School of Science, Minzu University of China, Beijing 100081, China; NHC Key Laboratory of Biosafety, National Institute for Viral Disease Control and Prevention, Chinese Center for Disease Control and Prevention, Beijing 102206, China; Research Unit of Adaptive Evolution and Control of Emerging Viruses, Chinese Academy of Medical Sciences, Beijing 102206, China; Institute of Pathogenic Organism, Shenzhen Center for Disease Control and Prevention, Shenzhen 518055, China; Respiratory Department , Shenzhen Children's Hospital, Shenzhen 518038, China; Institute of Pediatrics, Shenzhen Children's Hospital, Shenzhen 518038, China; Shenzhen Engineering Laboratory of Phosphorene and Optoelectronics; International Collaborative Laboratory of 2D Materials for Optoelectronics Science and Technology of Ministry of Education; Shenzhen Institute of Translational Medicine; Department of Otolaryngology, Shenzhen Second People's Hospital; the First Affiliated Hospital; Institute of Microscale Optoelectronics, Shenzhen University, Shenzhen 518060, China

**Keywords:** surface plasmon resonance, CRISPR, SARS-CoV-2, Omicron variant

## Abstract

The outbreak of the COVID-19 pandemic was partially due to the challenge of identifying asymptomatic and presymptomatic carriers of the virus, and thus highlights a strong motivation for diagnostics with high sensitivity that can be rapidly deployed. On the other hand, several concerning SARS-CoV-2 variants, including Omicron, are required to be identified as soon as the samples are identified as ‘positive’. Unfortunately, a traditional PCR test does not allow their specific identification. Herein, for the first time, we have developed MOPCS (Methodologies of Photonic CRISPR Sensing), which combines an optical sensing technology-surface plasmon resonance (SPR) with the ‘gene scissors’ clustered regularly interspaced short palindromic repeat (CRISPR) technique to achieve both high sensitivity and specificity when it comes to measurement of viral variants. MOPCS is a low-cost, CRISPR/Cas12a-system-empowered SPR gene-detecting platform that can analyze viral RNA, without the need for amplification, within 38 min from sample input to results output, and achieve a limit of detection of 15 fM. MOPCS achieves a highly sensitive analysis of SARS-CoV-2, and mutations appear in variants B.1.617.2 (Delta), B.1.1.529 (Omicron) and BA.1 (a subtype of Omicron). This platform was also used to analyze some recently collected patient samples from a local outbreak in China, identified by the Centers for Disease Control and Prevention. This innovative CRISPR-empowered SPR platform will further contribute to the fast, sensitive and accurate detection of target nucleic acid sequences with single-base mutations.

## Teaser

This paper presents an unprecedented SARS-CoV-2 variants gene detection platform MOPCS(Methodologies of Photonic CRISPR Sensing), which achieved high specificity and sensitivity at the same time by the innovative crossover of the unique sequence-specific recognizing ability of the CRISPR/Cas system and the superior sensitivity of the surface plasmon resonance sensing technique.

## INTRODUCTION

Severe acute respiratory syndrome coronavirus 2 (SARS-CoV-2) is rampaging across the world, and researchers are struggling to elucidate the mechanism of this disease and search for possible cures [1–4]. On the other hand, rapid, specific and sensitive gene detection methods are also required as the Omicron variant has become the most concerning variant [5]. As it stands, positive SARS-CoV-2 samples are still mainly tested by polymerase chain reaction (PCR), and identifying the variant types relies on gene sequencing [6]. However, these techniques are laborious, costly and time-consuming, requiring complex reactions using multiple reagents, skillful operators and expensive equipment. More importantly, the typical method only allows for the general detection of SARS-CoV-2 and not of specific strains. Therefore, based on the huge amount of gene information regarding variants that gene sequencing has built, new techniques can circumvent those limitations and promote the development of a fast and sensitive gene-detecting platform that discriminates different variants in a single step.

The clustered regularly interspaced short palindromic repeat (CRISPR) system is a well-known microbial natural adaptive immune system and was developed as a revolutionary genomic editing tool [7]. The widely known mechanism of CRISPR technology is CRISPR-associated (Cas) nuclease combined with a chimeric guide RNA (gRNA). Such a complex can bind to a gene locus with protospacer-adjacent motif (PAM), and recognize and cleave a site-specific nucleotide sequence [8]. This mechanism has been widely applied in gene therapies [9,10]. At the same time, gene-detecting methodologies that use different types of Cas nucleases have been developed and used widely. For example, Specific High-sensitivity Enzymatic Reporter unLOCKing (SHERLOCK) is a method employing Cas12 or Cas13 to detect preamplified DNA or RNA sequences [11]. Other methods like one-HOur Low-cost Multipurpose highly Efficient System (HOLMES) [12,13], HOLMESv2 [14] and CRISPR-Cas–only amplification network (CONAN) [15] are demonstrating the advantages of high specificity and flexibility. Such techniques have progressively been applied in different clinical scenarios: the detection of bacteria [16], diagnosis of hereditary disease [17], screening of viruses [18], etc.

Emerging diagnostic platforms based on CRISPR/Cas are reported to target SARS-CoV-2 with a combination of viral purification, amplification and detection processes [19–22]. These diagnostic methods are making SARS-CoV-2 testing more available in different application scenarios. However, the CRISPR-based diagnoses above rely on the amplification of sequences, leading to an increased need for experimental time [23], complicated devices and higher costs. Recently, researchers have attempted to avoid the use of polymerase-mediated amplification by improving the sensitivity of detection systems, like droplet microfluidics [24] and the design of modular catalytic hairpin assembly circuits [15,25]. Another way to thus improve the sensitivity of the detection system is by combining CRISPR with different platforms for enhanced signals. Kim *et al*. developed a CRISPR-based surface-enhanced Raman scattering (SERS) assay for genomic DNA, with a limit of detection (LOD) of ∼8–14 fM [26]. Hajian *et al*. also reported a graphene field-effect transistor coupling with the CRISPR/dCas9 system, as a specific capturer for specific exons of genomic DNA related to inherited disease [17].

Surface plasmon resonance (SPR)-sensing technology is a well-known versatile technique within the field of optical sensing platforms [27,28]. It is widely used in research into molecular interactions, including antigen–antibody [29], drug–target [30], protein-nucleic acid [31], protein–protein [32] and protein–lipid [33], and works by monitoring the refractive index change on the sensor surface caused by the surface change of weight. Our group is persistently making efforts with regard to SPR-sensing technologies for specific nucleic acid sensing [34]. More recently, we pioneered the combination of the CRISPR/Cas system with the SPR technique to establish a CRISPR-empowered SPR diagnostic platform. However, in the previous study [35], dCas9 protein and sgRNA had to be immobilized onto the chip in advance, making the preparation processes of the sensor complicated and time-consuming. Moreover, this previous system did not show the abilities of CRISPR to distinguish single mutated site in gene sequences.

To date, B.1.617.2 (Delta, originally discovered in India) and B.1.1.529 (Omicron, originally discovered in South Africa) are the two main SARS-CoV-2 subtypes of concern, and less protection from Omicron than Delta (when it came to the mainly used vaccines, Pfizer-BioNTech BNT162b2 and Moderna mRNA-1273) was implied [36]. There are multiple mutations on the S gene of both Delta and Omicron variants, and some of them are unique among the major variants (Alpha, Beta, Delta and Omicron) [37]. These mutations are the main reasons for the appearing variants of SARS-CoV-2 to escape the protection of vaccination and cause more false-negative results of gene detection, thereby wasting more social and medical resources. Although identification of variants through specialized sequencing centers is useful [6], many areas in developing countries are short of such resources. Furthermore, the delayed reports are not fulfilling the need for prompt tracking of SARS-CoV-2 variants. Henceforth, there is an urgent need for sensitive and specific diagnostic platforms for SARS-CoV-2 variants.

**Figure 1.**
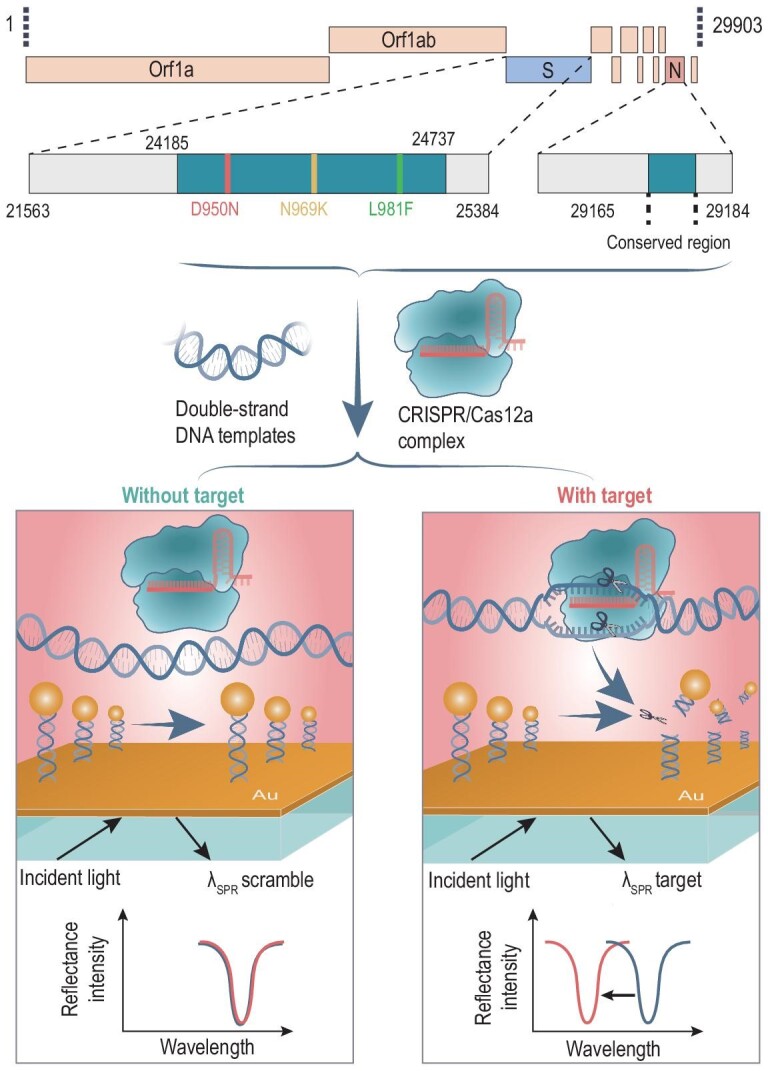
Scheme of MOPCS in this study. Within the whole genome sequence of SARS-CoV-2, a highly conserved region of N sequence was selected to detect the positive samples, and a region of S sequence with three featured mutation sites of Delta, Omicron and BA.1 variants was selected to distinguish the variants of positive samples. After the RNA sequences were extracted, purified and reverse transcribed into double-strand DNA templates, the templates were mixed with Cas12a-crRNA complexes. crRNAs were designed to target the conserved region, D950N, N969K and L981F mutation sites. After loading onto the SPR chips with pre-immobilized ssDNA reporter, only if the DNA templates contained the exact same sequences as the crRNA could the Cas12a be activated and the ssDNA reporter be trans-cleaved, inducing a changed (decreased) SPR wavelength.

Herein, we demonstrate the synergistic power of the highly sensitive SPR sensor and the mutation site-specific recognizing ability of the CRISPR/Cas12a system (Fig. 1), producing a biosensor that correctly reports the concentration of currently concerning variants of SARS-Cov-2 in both fake viruses and clinical samples. We name this sensing platform ‘MOPCS’ (Methodologies of Photonic CRISPR Sensing). In this study, we utilized the lateral (trans-) cleavage properties of the CRISPR/Cas12a system, therefore only gold-nanoparticle linked single-strand DNAs have to be immobilized on the chip in advance, which makes the preparation of the chips much easier than before. We fully utilized the single-site mutation distinguishing power of the CRISPR/Cas system to discern subtypes of SARS-CoV-2 as soon as the ‘positive’ signals came out. By targeting these unique mutated sites, we distinguished specific target variants in the samples in this study, even for BA.1, a subtype of Omicron, from ordinary Omicron samples. The advantages of MOPCS can be categorized as: (i) the SPR technique has high sensitivity when it comes to detecting amplification-free samples (LOD: 15 fM); (ii) the CRISPR/Cas12a system correctly distinguishes specific mutation sites and reports which variant the samples belong to. Above all, this novel CRISPR-empowered SPR detection can facilitate precise, stable, sensitive and reliable gene analysis of clinical samples with SARS-Cov-2 variants. This study has proved that there are possibilities with regard to combining CRISPR and SPR techniques, and consolidated further developments in this new nucleic acid detecting strategy.

## RESULTS AND DISCUSSION

### Bioinformatic analysis and selection of SARS-CoV-2 target regions

The MOPCS platform is capable of universal SARS-CoV-2 detection, by using crRNA that targets the same region on the N (nucleoprotein) gene of the Centers for Disease Control and Prevention in United States (US CDC) assay of qPCR [38]. Moreover, due to the high specificity of CRISPR technology, MOPCS can recognize featured single mutated sites of the emerging variants, for example, we demonstrate the capability of this platform to discern B.1.617.2 (Delta), B.1.1.529 (Omicron) and BA.1 variants in this study. For the Delta variant, the featured mutation of D950N (24410 G > A) was selected as the target site, because this mutation mapped to the trimer interface, suggesting that this mutation may contribute to the regulation of spike protein dynamics [39]. Furthermore, the Omicron variant and its subtype BA.1 contains mutation sites including N969K (24469 T > A, both Omicron and BA.1) and L981F (24503 C > T, BA.1 only) [40], and those sites were selected to distinguish them according to variants using MOPCS (Fig. 1).

### SHERLOCK assay that specifically identifies SARS-CoV-2 variants

**Figure 2.**
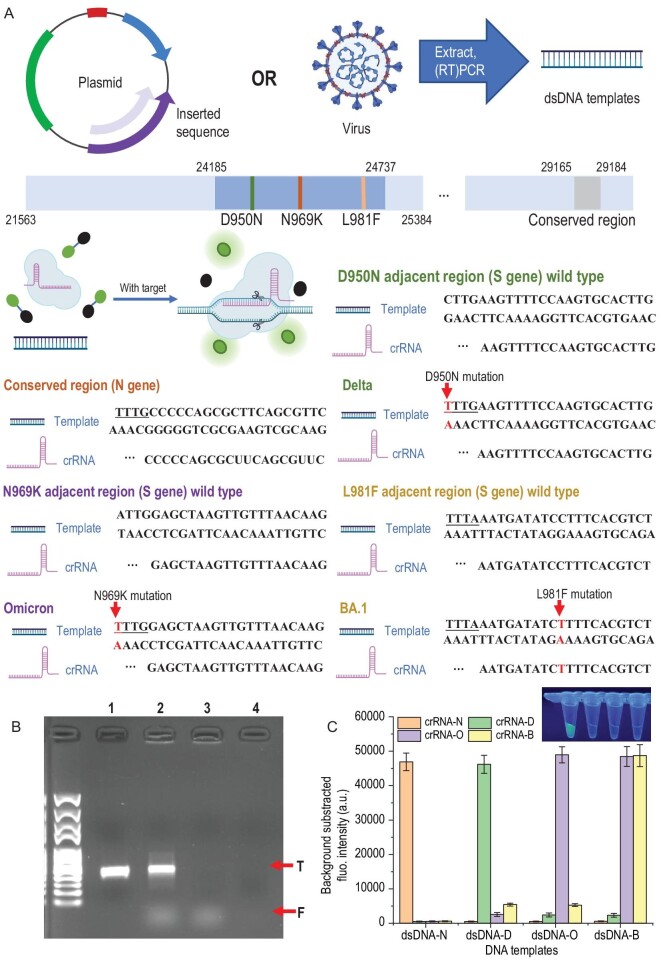
DNA templates, design of crRNAs and the verification of specificity. (A) dsDNA templates were acquired by amplifying certain inserted sequences in the plasmids or extracting RNA from the virus and performing RT-PCR. SHERLOCK assay was performed by mixing Cas12a protein, crRNA, DNA template and FAM-BHQ probes and incubating at 37°C for 30 min. Only if the crRNA and DNA were paired would the trans-cleavage happen and freed FAM generate fluorescence. Several mutation sites on S gene sequence and a highly conserved region on N gene sequence were selected as the targets. For D950N and N969K mutation sites, representing Delta and Omicron variants respectively, TTTV PAM sequences are formed and therefore they have a much higher affinity with the Cas12a protein compared to wild-type sequences. For L981F, which represents the Omicron-BA.1 variant, crRNA was designed according to the mutation sequence. (B) Verification of successful trans-cleavage and cis-cleavage was performed using agarose gel electrophoresis. Lane 1, 100 nM DNA template (N gene) only; Lane 2, the 100 nM DNA template performed a SHERLOCK assay, cis-cleavage (T band) and freed FAM coursed by trans-cleavage (F band) were observed; Lane 3, the 1 nM DNA template performed a SHERLOCK assay, although the band of DNA was not observed because of the low loading amount—trans-cleavage (F band) was also observed; Lane 4, 0.01 nM DNA template performed SHERLOCK assay—neither of the bands was observed because the loading amount was too low and no FAM was freed by trans-cleavage. (C) Specificity of the crRNA-dsDNA pairs was performed by cross-reactions between each crRNA and dsDNA template (10 nM). Only right paired reactions were observed with strong fluorescence signals: dsDNA-N and crRNA-N; dsDNA-D and crRNA-D; dsDNA-O and crRNA-O; dsDNA-B and crRNA-O/crRNA-B (because BA.1 is one subtype of Omicron). The difference between positive reactions (strong fluorescence) and negative reactions (transparent) is also presented.

MOPCS is based on the SHERLOCK assay that uses the CRISPR/Cas12a system to detect specific target gene sequences. Therefore, we performed SHERLOCK assays to confirm the specificity of each designed crRNA to their target sequences prior to the on-device detection of MOPCS. As shown in Fig. 2, five recombinant pUC57 plasmids were prepared for the dsDNA templates for the CRISPR/Cas12a system, providing regions of the wild-type N sequence, wild-type S sequence, S sequence of Delta variant, S sequence of Omicron variant and S sequence of BA.1 variant, respectively. The detailed inserted sequences can be found in Table S2, and the successful construction of variants was verified using Sanger Sequencing (Fig. S1 in the online supplementary data). After being amplified using PCR (primers are shown in Table S1), the concentrations of linear dsDNA products were measured using NanoDrop (ThermoFisher Scientific) and stored at −80°C until use.

SHERLOCK assay was performed using the dsDNA templates and each crRNA to verify the specificity (Fig. 2). As shown in Fig. 2A, there are two ways to acquire dsDNA templates used in this study. One is inserting target sequences, including a conserved sequence of N gene, and wild type, Delta, Omicron, or Omicron-BA.1 sequences in the pUC57 plasmids, and amplifying the desired regions. The other way is extracting RNA from the virus and performing Reverse transcription Polymerase Chain Reaction (RT-PCR) to acquire the target regions. SHERLOCK assay was performed by mixing Cas12a protein, crRNA, DNA template and Carboxyfluorescein-Black Hole Quencher (FAM-BHQ) probes and incubating at 37°C for 30 min. Several mutation sites on the S gene sequence and a highly conserved region on the N gene sequence were selected as the targets. For D950N and N969K mutation sites, representing Delta and Omicron variants respectively, TTTV PAM sequences are formed and therefore they have a much higher affinity with the Cas12a protein than the wild-type sequences. For L981F, which represents the Omicron-BA.1 variant, crRNA was designed according to the mutation sequence.

Verification of successful trans-cleavage and cis-cleavage was performed using agarose gel electrophoresis. As shown in Fig. 2B: lane 1, 100 nM DNA template (N gene) only; lane 2, 100 nM DNA template performed SHERLOCK assay, cis-cleavage (T band) and freed FAM coursed by trans-cleavage (F band) were observed; lane 3, 1 nM DNA template performed SHERLOCK assay, although the band of DNA was not observed because of the low loading amount—trans-cleavage (F band) was also observed; lane 4, 0.01 nM DNA template performed SHERLOCK assay, neither of the bands was observed because the loading amount was too low and no FAM was freed by trans-cleavage.

Specificity of the crRNA-dsDNA pairs was performed by cross-reactions between each crRNA and dsDNA template (Fig. 2C). Only right paired reactions were observed with strong fluorescence signals: dsDNA-N and crRNA-N; dsDNA-D and crRNA-D; dsDNA-O and crRNA-O; dsDNA-B and crRNA-O/crRNA-B (because BA.1 is one subtype of Omicron). The difference between positive reactions (strong fluorescence) and negative reactions (transparent) is also presented.

Through the results above, highly specific trans-cleavage of crRNA targeting variants of sequences from SARS-CoV-2 was verified, however, DNA templates in low concentration (<0.01 nM) cannot be determined. Therefore, in the following experiments, sensors based on SPR were applied to improve the sensitivity.

### MOPCS on-device measurement

**Figure 3.**
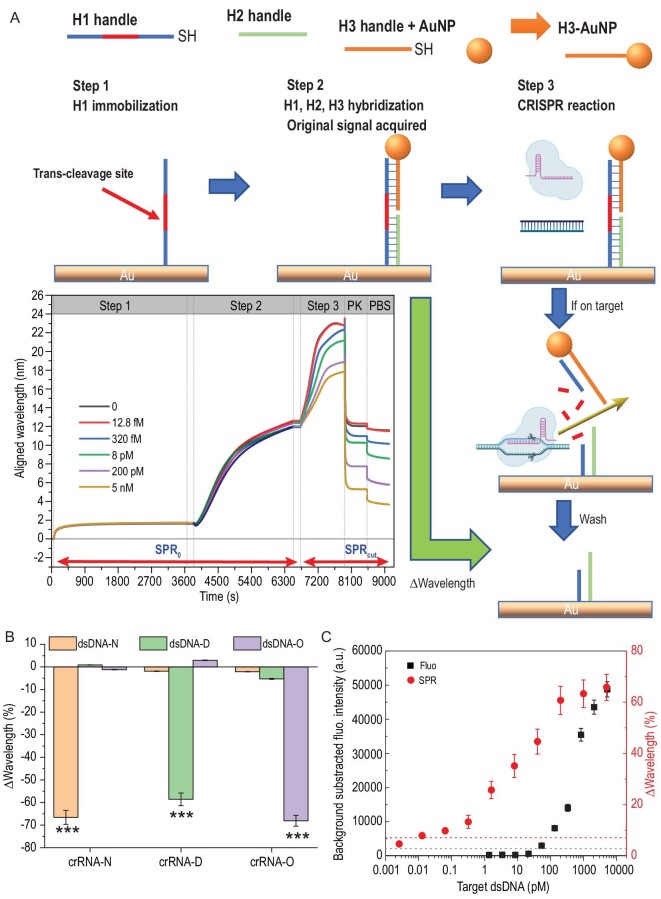
On-device measurement. (A) Scheme of the workflow. Three single-strand DNA handles were designed and synthesized: H1, a long handle containing a sequence for trans-cleavage of activated Cas12a; H2, a short handle with a complementary sequence of part of H1; H3, another short handle with a complementary sequence of the other part of H1, and it was linked to Au nanoparticles by using the thiol-free freezing method. On-device, H1 was firstly immobilized onto the Au surface of SPR chips. After short rinsing, H2 and H3-AuNP handles were added to the flow cell, and a double-strand DNA structure was formed. During the CRISPR detecting process, Cas12a protein was activated if the DNA template was the target of crRNA and would thus resulted in trans-cleave H1, followed by the release of Au nanoparticles. By washing the surface to remove non-specific absorption of a substance like Cas12a protein, the SPR signal could be determined. The higher concentrations of DNA templates caused an increase in final shifted wavelengths from the endpoint of H2 + AuNPs@H3. (B) Specificity of on-device measurement was verified by cross-reaction of each crRNA and dsDNA template. (C) A comparison between the fluorescent signal and SPR signal, testing different concentrations of target dsDNA template (*n* = 3, ^***^P < 0.001).

Based on the specificity of crRNA-target sequences proved above, on-device measurement was performed to further improve sensitivity and lower the LOD to meet the requirement for the detection of unamplified samples from patients. Here, we used H1-, H2- and AuNP@H3-handle-assembled reporters because, on one hand, this design can be programmed to link other nanomaterials on the H3 handle, and on the other hand, the partly double-strand structure can maintain a more stable distance between the chip surface and the AuNP, to offer a more stable SPR signal [41]. The on-device workflow is described in Fig. 3A and Fig. S2, and stated below. Firstly, three single-strand DNA reporters were prepared: H1, a long handle containing a sequence for trans-cleavage of activated Cas12a; H2, a short handle with a complementary sequence of part of H1; H3, another short handle with a complementary sequence of the other part of H1—this was linked to Au nanoparticles by using the thiol-free freezing method [20]. On-device, started with Phosphate Buffered Saline (PBS) rinsing on the chip (wavelength = λ_0_), H1 was then immobilized onto the Au surface of SPR chips. After short rinsing, H2 and AuNP@H3 handles were added to the flow cell, and a double-strand DNA structure was formed (wavelength = λ_init_). After reacting with the matrix containing the Cas12a, crRNA and DNA templates, Cas12a protein would be activated if the DNA template was the target of crRNA, and thus resulted in trans-cleave H1, followed by the release of Au nanoparticles. By washing the surface to remove non-specific absorption of a substance like Cas12a protein, the final SPR signal (wavelength = λ_end_) can be determined. The ΔWavelength, which is calculated as SPR_cut_/SPR_0_ (where SPR_cut_ = λ_init__−_λ_end_, and SPR_0_ = λ_init_ − λ_0_) is the result that reflects the concentration of target dsDNA templates. The more target sequences that exist in the reaction, the more Cas12a can be activated, thus the more H1 handle would be cleaved in a certain time. Therefore, the ΔWavelength signal can reflect the concentration of target sequences. Because the steps before CRISPR can be prepared in advance, the total test time is 6720∼9000 s, in other words, it can be <38 min.

Next, the specificity of on-device measurement was verified by cross-reaction of each crRNA and dsDNA template (Fig. 3B). As a result, right-paired crRNA and dsDNA can cause a significantly higher ΔWavelength (N: 66.6% ± 3.1%, D: 58.6% ± 3.1%, O: 66.6% ± 2.4%) compared to any wrong paired groups. The specificity was cross-proved with the results of fluorescent methods (Fig. 2).

Furthermore, a comparison between fluorescent signal and SPR signal, testing different concentrations of target dsDNA templates, was performed (Fig. 3C). Firstly, the positive threshold was defined as the average of the blank signals plus triple its standard deviation, which is 2117 a.u. for the fluorescent measure (black dashed line) and 7.11% for ΔWavelength signal (red dashed line). Any signal above the positive threshold indicates the existence of target sequences. Therefore, the LOD for the fluorescent method and MOPCS is 50 pM and 15 fM, respectively. This means that the sensitivity of MOPCS is >1000-fold higher than the typical fluorescent method of CRISPR diagnosis for target gene sequences.

### Performance of the MOPCS diagnostic device and validation with clinical samples

The ultimate purpose of MOPCS is to practically detect clinical samples with target nucleic acids. We tested 30 samples from SARS-CoV-2 patients identified as having the Omicron variant; this was already determined using qPCR and Sanger sequencing by the Shenzhen Center for Disease Control and Prevention (China). From healthy people, 30 samples were also used as the negative control.

**Figure 4.**
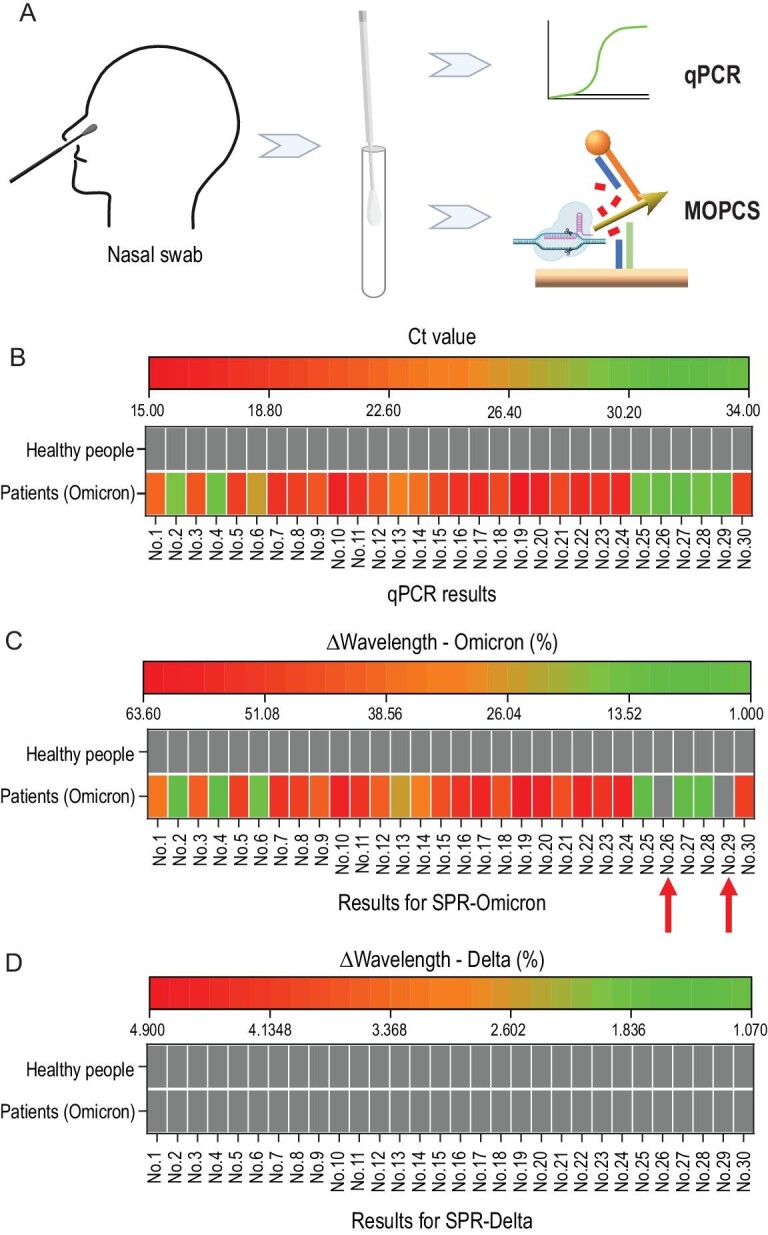
Validation of MOPCS with clinical samples. (A) Nasal swabs were obtained from patients, and the virus was deactivated and followed by RT-qPCR or MOPCS. (B) qPCR results for 30 patients (Omicron variant) and healthy people. (C) MOPCS for detection of the N969K mutation site (only in Omicron variant) were performed using the same samples of qPCR. Positive SPR signals for two of the patients (arrowed) were not detected (false negatives). (D) MOPCS for detection of the D950N mutation site (only in Delta variant) were performed using the same samples above, and no positive result was shown.

As shown in Fig. 4, nasal swabs were obtained from patients. The virus was deactivated and followed by MOPCS, and compared with the results of qPCR. The qPCR results (Fig. 4B) for 30 patients were all positive (Ct value < 40), and the results for healthy people were all negative (not reaching the threshold within 40 circles of amplification). MOPCS for detection of the N969K mutation site (only exists in the Omicron variant) was performed using the same samples. Similar to the results of qPCR, no positive result was shown in the healthy control group. However, positive SPR signals for two of the patients (Fig. 4C, arrowed) were not detected, which was a false-negative result. The accuracy of MOPCS in this test was 93.3% (28 out of 30 patients were detected). Because no amplification step was involved, this accuracy is relatively high. Moreover, MOPCS for detection of the D950N mutation site (only in the Delta variant) was performed using the same samples above, and no positive result had been shown (Fig. 4D), indicating that MOPCS could not only detect the positive samples but also distinguish the variant of SARS-Cov-2. Obviously, if general detection of SARS-Cov-2 is desired, MOPCS can perform with only crRNA targeting the conserved sequence of the N gene.

## CONCLUSION

As SARS-CoV-2 variants emerge, the control of the ongoing COVID-19 pandemic requires a gene-detecting platform with both high sensitivity and specificity. The streamlined workflow and flexible design of MOPCS—a label-free and rapid CRISPR-empowered SPR technique—were developed for detecting different variants of SARS-CoV-2. In this study, featured mutation sites of the Delta, Omicron and Omicron-BA.1 variants were selected, and the specificity of the designed crRNAs was verified. To resolve the problem of the low sensitivity of typical CRISPR assays, we developed a combined assay of CRISPR and SPR to achieve 1000-fold sensitivity; the LOD of MOPCS reached 15 fM without the process of preamplification of the samples, and the on-device test time was ∼38 min. Finally, we tested positive samples from SARS-CoV-2 patients to validate the accuracy and the ability to distinguish variants by MOPCS, and no false-positive results occurred. We have built the rudimentary methods for combining the utilities of CRISPR and SPR, and hopefully it will further develop in several aspects. In future studies, this system could be improved by functionalizing with nanomaterials to further improve the SPR signal response, and integrating with microfluidic technology for multiplex gene detection. Above all, with the integrated sequence-specific recognizing ability of the CRISPR/Cas system and the high sensitivity of the SPR sensor, we provide a novel sensing methodology for precise, stable, sensitive and reliable gene analysis of clinical samples.

## METHODS

### Sample preparation

As the dsDNA templates—plasmids containing wild-type or mutated N gene or S gene sequences (Table S2)—were also synthesized by Sangon Biotech (Shanghai) Co. Ltd., the Delta, Omicron and BA.1 mutation sites were verified by Sanger sequencing (Fig. S1). Further, samples from 30 patients confirmed ‘positive’ by qPCR previously were collected and MOPCS detection was performed.

### On-device reactions

H1-, H2-, H3-handle (Table S1) assembled reporters were used on the SPR device. Firstly, H3 DNA solution (5 μL, 100 μM; shown in Table S1) was added to 100 μL of AuNP (15 nm in diameter) solution and mixed via a brief vortex. After being at −20°C for 2 h, the solution was thawed at room temperature (RT). Finally, the mixture was centrifuged at 12 000 rpm for 20 min and the supernatant was removed. The pellet was washed three times with PBS to remove free DNA. The conjugate was redispersed in PBS for further use.

For the SPR measurement, thiolate H1 ssDNA handle (Table S1) was firstly dissolved in 100 mM PBS buffer solution (10 mM tris(2-carboxyethyl)phosphine (TCEP) in PBS, pH 7.4) for 30 min. After the SPR chip was rinsed by PBS for 1 min, thiolate ssDNA solution was introduced into the Teflon sample chamber via a capillary tube for over 60 min at 37°C. Subsequently, the SPR chip was rinsed with PBS for 3 min and further incubated with the H2 handle and AuNPs@H3 handle described above, for 45 min at 37°C. After rinsing with PBS buffer for 3 min, the chip was ready to use for detecting the samples. The reactions of SHERLOCK assay (100 nM Cas12a New England Biolabs (NEB), 100 nM crRNA, 1 × NEB 2.1 buffer (NEB) and target dsDNA sequences at desired concentrations) were introduced into the chamber and incubated for 20 min at 37°C. Subsequently, Proteinase K (1:1000) in 1 × NEB 2.1 buffer was introduced into the chamber and incubated for 10 min at 37°C to remove the non-specific binding of Cas12a protein on the SPR chip. Finally, the SPR signal was acquired after rinsing with PBS briefly. The change in SPR signal was used to quantify the target dsDNA sequences in SHERLOCK reactions. The SPR response was monitored in real time for further analysis. All the SPR curves were smoothed using the Savitzky-Golay method.

### DATA AVAILABILITY

The data that support the findings of this study are available from the corresponding authors upon reasonable request.

## Supplementary Material

nwac104_Supplemental_File
