# Supplementary material for: A CRISPR/Cas12a-empowered surface plasmon resonance platform for rapid and specific diagnosis of the Omicron variant of SARS-CoV-2
Source: Natl Sci Rev. 2022 Jun 3;9(8):nwac104. doi: 10.1093/nsr/nwac104 (PMC9385456; doi:10.1093/nsr/nwac104)
Supplement: nwac104_Supplemental_File [file nwac104_supplemental_file.docx]

**A CRISPR/Cas12a empowered surface plasmon resonance platform for rapid and specific diagnosis of the Omicron variant of SARS-CoV-2**

***Supplementary Information***

Zhi Chen ^1,#^, Jinfeng Li ^1,2,#^, Tianzhong Li ^1,2^, Taojian Fan ^1^, Changle Meng ^1^, Chaozhou Li ^1^, Jianlong Kang ^1^, Luxiao Chai ^1^, Yabin Hao ^1,5^, Yuxuan Tang ^1,6^, Omar A. Al-Hartomy ^7^, Swelm Wageh ^7^, Abdullah G. Al-Sehemi ^8,9^, Zhiguang Luo ^10^, Jiangtian Yu ^2^, Yonghong Shao ^11^, Defa Li ^12^, Shuai Feng ^13^, William J. Liu ^14, 15^, Yaqing He ^16,*^, Xiaopeng Ma ^4,*^, Zhongjian Xie ^3,*^, Han Zhang ^1,*^

^1^Shenzhen Engineering Laboratory of Phosphorene and Optoelectronics; International Collaborative Laboratory of 2D Materials for Optoelectronics Science and Technology of Ministry of Education; Shenzhen Institute of Translational Medicine; Department of Otolaryngology, Shenzhen Second People's Hospital; the First Affiliated Hospital; Institute of Microscale Optoelectronics, Shenzhen University, Shenzhen 518060, China;

^2^Shenzhen International Institute for Biomedical Research, Shenzhen 518116, Guangdong, China;

^3^Institute of Pediatrics, Shenzhen Children’s Hospital, Shenzhen 518038, China;

^4^Department of Respiratory, Shenzhen Children’s Hospital, Shenzhen 518038, China;

^5^Shenzhen Han’s Tech Limited Company. Shenzhen 518000, China;

^6^Shenzhen Metasensing Tech Limited Company. Shenzhen 518000, China;

^7^Department of Physics, Faculty of Science, King Abdulaziz University, Jeddah 21589, Saudi Arabia;

^8^Research Center for Advanced Materials Science (RCAMS), King Khalid University, Abha 61413, Saudi Arabia;

^9^Department of Chemistry, College of Science, King Khalid University, Abha 61413, Saudi Arabia;

^10^Zhongmin (Shenzhen) intelligent ecology Co., Ltd., Shenzhen 518055, China;

^11^Key Laboratory of Optoelectronic Devices and Systems of Ministry of Education and Guangdong Province, College of Physics and Optoelectronic Engineering, Shenzhen University, Shenzhen 518060, China;

^12^Department of Laboratory Medicine, Shenzhen Children’s Hospital, Shenzhen 518038, China;

^13^Optoelectronics Research Center, School of Science, Minzu University of China, Beijing 100081, China;

^14^NHC Key Laboratory of Biosafety, National Institute for Viral Disease Control and Prevention, Chinese Center for Disease Control and Prevention, Beijing 102206, China;

^15^Research Unit of Adaptive Evolution and Control of Emerging Viruses, Chinese Academy of Medical Sciences, Beijing 102206, China；

^16^Shenzhen Center for Disease Control and Prevention, Shenzhen 518055, China

^*^**Corresponding authors**. E-mails: [heyaqing1019@126.com](mailto:heyaqing1019@126.com); maxiaopeng@126.com; zjxie@siitm.org.cn; [hzhang@szu.edu.cn](mailto:hzhang@szu.edu.cn)

^#^Equally contributed to this work.

**METHODS**

**Materials and reagents**

Lba Cas12a and 10 × NEBuffer 2.1 (0.5 M NaCl, 0.1 M Tris-HCl, 0.1 M MgCl_2_, and 100 µg/mL BSA, pH 7.9) were obtained from New England Biolabs (Ipswich, MA, USA). RNase inhibitors, RNase-free water, and DNA Marker were obtained from Takara Biotech. Inc. (Dalian, China). Proteinase K, tris(2-carboxyethyl)-phosphine hydrochloride (TCEP), and polyethylene glycol sorbitan monolaurate (Tween-20) were purchased from Sigma-Aldrich (St. Louis, USA). All DNA oligonucleotides and HPLC-purified crRNAs, (Table S1) were synthesized and purified from Sangon Biotech Co., Ltd. (Shanghai, China). All chemical reagents were of analytical grade, and RNase-free water was used throughout this study.

**Instrumentation**

All SPR measurements were carried out with a home-built wavelength-interrogated SPR system (1,2). A Cary Eclipse fluorescence spectrophotometer (Agilent Technologies, Palo Alto, CA) was employed to monitor the fluorescence spectra). A NanoDrop 1000 spectrophotometer (Thermo Scientific, USA) was used to quantify DNA suspensions. The gel electrophoresis analysis was performed by an electrophoresis analyzer (Bio-Rad, USA) and imaged on a ChemiDoc XRS system (Bio-Rad, USA).

**Bioinformatic analysis of SARS-CoV-2 genomes and crRNA design**

The 29903nt- complete genome of SARS-CoV-2 was downloaded from NCBI (NCBI NC_045512.2). For universal SARS-CoV-2 detection, we first designed primers targeting the nucleocapsid (N) genes that amplify regions (29019-29344, 325bp) that overlap the US CDC assay (3,4). Further, crRNA targeting a 20-nt sequence (29165-29184) narrowed from the amplicon of the N region was also synthesized and referred to as crRNA-N.

For detecting the variants, part of the spike (S) gene (24192-24643, 452bp) was selected to analyze because there are several mutations related to B.1.617.2 (Delta), B.1.1.529 (Omicron), and BA.1 variant in this region. For the Delta variant, mutation of D950N (24410 G ˃ A) was selected as the target because this mutation was not found in the original sequence or other mutations (Omicron and BA.1), it is unique in the Delta variant. For presenting the ability to distinguish the Omicron variant and its subtype BA.1, we tested mutations on N969K (24469 T ˃ A, both Omicron and BA.1) and L981F (24503 C ˃ T, BA.1 only). Based on the mutation sites, specific crRNAs are synthesized and referred to as crRNA-D (Delta), crRNA-O (Omicron), and crRNA-B (BA.1), respectively.

There are two parts of Cas12a crRNAs: the universal scaffold region (UAAUUUCUA CUAAGUGUAGAU) for Cas12a protein recognition and binding, and a customized region added to the 3’ end of the scaffold that provides the specificity to the target sequences (the same sequence to the region following a TTTV PAM sequence). The designing principle is consistent with the ‘CRISPR-SHERLOCK’ method (5) published by Feng Zhang et al. All the crRNA and primer sequences are provided by Sangon Biotech (Shanghai) Co., Ltd. and listed in *Table S1*.

**Sample preparation**

As the dsDNA templates, plasmids containing wild-type or mutated N gene or S gene sequences (*Table S2*) were also synthesized by Sangon Biotech (Shanghai) Co., Ltd. The Delta, Omicron, and BA.1 mutation sites were verified by Sanger sequencing (*Figure S1*). Further, samples from thirty patients confirmed ‘positive’ by qPCR previously were collected and performed MOPCS detection.

**SHERLOCK assay**

The specificity of crRNAs to different sequences from different variants of SARS-CoV-2 was validated by performing the SHERLOCK assay. In 10 μL of volumes using 100 nM Cas12a (NEB), 100 nM crRNA, 1× NEB 2.1 buffer (NEB), 500 nM single-stranded DNA (ssDNA) fluorescent quenched reporter (5’ 6-FAM/TTATTATT/BHQ-1 3’, Sangon), and 10 nM target dsDNA sequences of wild-type N gene, wild-type S gene, Delta S gene, Omicron S gene, or BA.1 S gene diluted in Rnase/Dnase-free water. Reactions were incubated at 37°C for 30 min, and real-time fluorescence was measured using a BioTek NEO HTS plate reader (BioTek Instruments) with readings every 2 min (excitation: 485 nm; emission: 528 nm). The cis-cleavage of sequences was further verified by electrophoresis on 2.5% agarose gel under 120V for 30 min.

**On-device reactions**

H1, H2, H3 handle (Table S1) assembled reporter was used on the SPR device. Firstly, H3 DNA solution (5 μL, 100 μM; shown in Table S1) was added to 100 μL of AuNP (15 nm in diameter) solution and mixed via a brief vortex. After placing at -20 °C for 2 h, the solution was thawed at room temperature (RT). Finally, the mixture was centrifuged at 12 000 rpm for 20 min and the supernatant was removed. The pellet was washed 3 times with PBS to remove free DNA. The conjugate was redispersed in PBS for further use.

For the SPR measurement, thiolate H1 ssDNA handle (Table S1) was firstly dissolved in 100 mM PBS buffer solution (10 mM TCEP in PBS, pH 7.4) was performed for 30 min. After the SPR chip was rinsed by PBS for 1 min, thiolate ssDNA solution was introduced into the Teflon sample chamber via a capillary tube over 60 min at 37°C. Subsequently, the SPR chip was rinsed with PBS for 3 min and further dealt with H2 handle and AuNPs@H3 handle described above, for 45 min at 37°C. After rinsing with PBS buffer for 3 min, the chip is ready to use for detecting the samples. The reactions of SHERLOCK assay (100 nM Cas12a (NEB), 100 nM crRNA, 1× NEB 2.1 buffer (NEB), and target dsDNA sequences at desired concentrations) was introduced into the chamber and incubated for 20 min at 37°C. Subsequently, Proteinase K (1:1000) in 1× NEB 2.1 buffer was introduced into the chamber and incubated for 10 min at 37°C to remove the non-specific binding of Cas12a protein on the SPR chip. Finally, the SPR signal was acquired after rinsing with PBS briefly. The change in SPR signal was used to quantify the target dsDNA sequences in SHERLOCK reactions. The SPR response was monitored in real-time for further analysis. All the SPR curves were smoothed using the Savitzky-Golay method.

**Statistics**

All measurements were performed in triplicate (n=3), and the data are displayed as mean ± standard deviation. Correlations were performed with linear regression to determine the goodness of ﬁt (Pearson’s correlation coefﬁcient, R2). For inter-sample comparisons, multiple pairs of samples were analyzed by a two-tailed t-test, and the resulting P values were adjusted for multiple hypothesis testing using Bonferroni correction. *P<0.05, **P<0.01, and ***P <0.001 indicate obvious statistical differences. All statistical analyses were performed using OriginPro (v.2019b).

**Table S1 Primers, crRNAs, and reporters used in this study**

| Name | Sequence (5’-3’) |
| --- | --- |
| N-primer-F | AATCTGCTGCTGAGGCTTCT |
| N-primer-R | GCTCTGTTGGTGGGAATGTTT |
| S-primer-F | TGTTAGCGGGTACAATCACTTCTGGTT |
| S-primer-R | AGTAGCAGCAAGATTAGCAGAA |
| crRNA-N | UAAUUUCUACUAAGUGUAGAUCCCCCAGCGCUUCAGCGUUC |
| crRNA-D | UAAUUUCUACUAAGUGUAGAUAAGTTTTCCAAGTGCACTTG |
| crRNA-O | UAAUUUCUACUAAGUGUAGAUGAGCTAAGTTGTTTAACAAG |
| crRNA-B | UAAUUUCUACUAAGUGUAGAUAATGATATCTTTTCACGTCT |
| FAM-reporter | FAM-TTATTATT-BHQ |
| H1 handle | SH-CTTTACTCAACttattattACGAACATCAGG |
| H2 handle | ataaGTTGAGTAAAG |
| H3 handle | SH-CCTGATGTTCGTaata |

**Table S2 Sequences of wild-type or mutated N gene or S gene in pUC57 plasmids.** The target 20nt- sequences for CRISPR reaction are **bolded**, PAM sequences (or their complementary strands) are underlined, and mutated sites are presented as lower-case letters and labelled as red.

| Name | Inserted Sequences (5’-3’) |
| --- | --- |
| N-gene-ori-plasmid | AGGCCAACAACAACAAGGCCAAACTGTCACTAAGAAATCTGCTGCTGAGGCTTCTAAGAAGCCTCGGCAAAAACGTACTGCCACTAAAGCATACAATGTAACACAAGCTTTCGGCTTGGGGACCAGGAACTAATCAGACAAGGAACTGATTACAAACATTGGCCGCAAATTGCACAATTTG**CCCCCAGCGCTTCAGCGTTC**TTCGGAATGTCGCGCATTGGCATGGAAGTCACACCTTCGGGAACGTGGTTGACCTACACAGGTGCCATCAAATTGGATGACAAAGATCCAAATTTCAAAGATCAAGTCATTTTGCTGAATAAGCATATTGACGCATACAAAACATTCCCACCAACAGAGCCTAAAAAGGACAAAAAGAAGAAGGCTGATGAAACTCAA |
| S-gene-ori-plasmid | TCTGCACTGTTAGCGGGTACAATCACTTCTGGTTGGACCTTTGGTGCAGGTGCTGCATTACAAATACCATTTGCTATGCAAATGGCTTATAGGTTTAATGGTATTGGAGTTACACAGAATGTTCTCTATGAGAACCAAAAATTGATTGCCAACCAATTTAATAGTGCTATTGGCAAAATTCAAGACTCACTTTCTTCCACAG**CAAGTGCACTTGGAAAACTT**CAAGATGTGGTCAACCAAAATGCACAAGCTTTAAACACG**CTTGTTAAACAACTTAGCTC**CAATTTTGGTGCAATTTCAAGTGTTTTA**AATGATATCCTTTCACGTCT**TGACAAAGTTGAGGCTGAAGTGCAAATTGATAGGTTGATCACAGGCAGACTTCAAAGTTTGCAGACATATGTGACTCAACAATTAATTAGAGCTGCAGAAATCAGAGCTTCTGCTAATCTTGCTGCTACTAAAATGTCAGAGTGTGTACTTGGACAATCAAAAAGAGTTGATTTTTGTGGAAAGGGCTATCATCTTATGTCCTTCCCTCAGTCAGCACCTCAT |
| S-gene-delta-plasmid | TCTGCACTGTTAGCGGGTACAATCACTTCTGGTTGGACCTTTGGTGCAGGTGCTGCATTACAAATACCATTTGCTATGCAAATGGCTTATAGGTTTAATGGTATTGGAGTTACACAGAATGTTCTCTATGAGAACCAAAAATTGATTGCCAACCAATTTAATAGTGCTATTGGCAAAATTCAAGACTCACTTTCTTCCACAG**CAAGTGCACTTGGAAAACTT**CAAaATGTGGTCAACCAAAATGCACAAGCTTTAAACACGCTTGTTAAACAACTTAGCTCCAATTTTGGTGCAATTTCAAGTGTTTTAAATGATATCCTTTCACGTCTTGACAAAGTTGAGGCTGAAGTGCAAATTGATAGGTTGATCACAGGCAGACTTCAAAGTTTGCAGACATATGTGACTCAACAATTAATTAGAGCTGCAGAAATCAGAGCTTCTGCTAATCTTGCTGCTACTAAAATGTCAGAGTGTGTACTTGGACAATCAAAAAGAGTTGATTTTTGTGGAAAGGGCTATCATCTTATGTCCTTCCCTCAGTCAGCACCTCAT |
| S-gene-omicron-plasmid | TCTGCACTGTTAGCGGGTACAATCACTTCTGGTTGGACCTTTGGTGCAGGTGCTGCATTACAAATACCATTTGCTATGCAAATGGCTTATAGGTTTAATGGTATTGGAGTTACACAGAATGTTCTCTATGAGAACCAAAAATTGATTGCCAACCAATTTAATAGTGCTATTGGCAAAATTCAAGACTCACTTTCTTCCACAGCAAGTGCACTTGGAAAACTTCAAGATGTGGTCAACCAAAtTGCACAAGCTTTAAACACG**CTTGTTAAACAACTTAGCTC**CAAaTTTGGTGCAATTTCAAGTGTTTTAAATGATATCCTTTCACGTCTTGACAAAGTTGAGGCTGAAGTGCAAATTGATAGGTTGATCACAGGCAGACTTCAAAGTTTGCAGACATATGTGACTCAACAATTAATTAGAGCTGCAGAAATCAGAGCTTCTGCTAATCTTGCTGCTACTAAAATGTCAGAGTGTGTACTTGGACAATCAAAAAGAGTTGATTTTTGTGGAAAGGGCTATCATCTTATGTCCTTCCCTCAGTCAGCACCTCAT |
| S-gene-BA.1-plasmid | TCTGCACTGTTAGCGGGTACAATCACTTCTGGTTGGACCTTTGGTGCAGGTGCTGCATTACAAATACCATTTGCTATGCAAATGGCTTATAGGTTTAATGGTATTGGAGTTACACAGAATGTTCTCTATGAGAACCAAAAATTGATTGCCAACCAATTTAATAGTGCTATTGGCAAAATTCAAGACTCACTTTCTTCCACAGCAAGTGCACTTGGAAAACTTCAAGATGTGGTCAACCAAAtTGCACAAGCTTTAAACACGCTTGTTAAACAACTTAGCTCCAAaTTTGGTGCAATTTCAAGTGTTTTA**AATGATATCtTTTCACGTCT**TGACAAAGTTGAGGCTGAAGTGCAAATTGATAGGTTGATCACAGGCAGACTTCAAAGTTTGCAGACATATGTGACTCAACAATTAATTAGAGCTGCAGAAATCAGAGCTTCTGCTAATCTTGCTGCTACTAAAATGTCAGAGTGTGTACTTGGACAATCAAAAAGAGTTGATTTTTGTGGAAAGGGCTATCATCTTATGTCCTTCCCTCAGTCAGCACCTCAT |


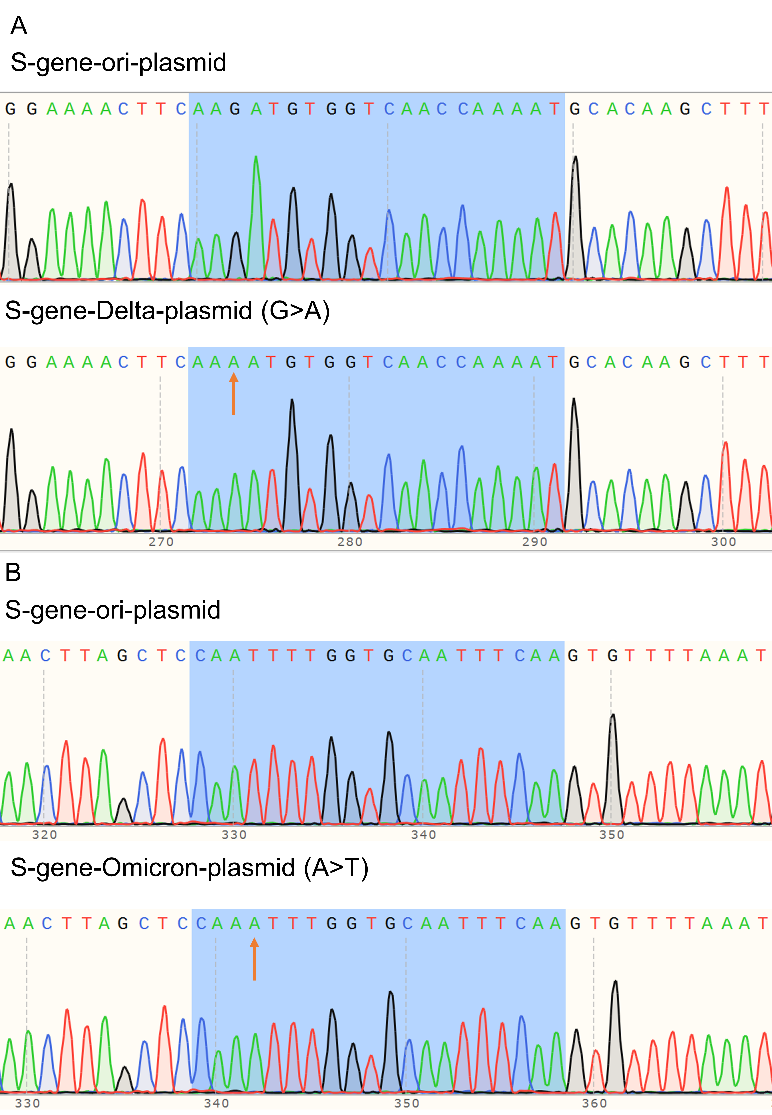


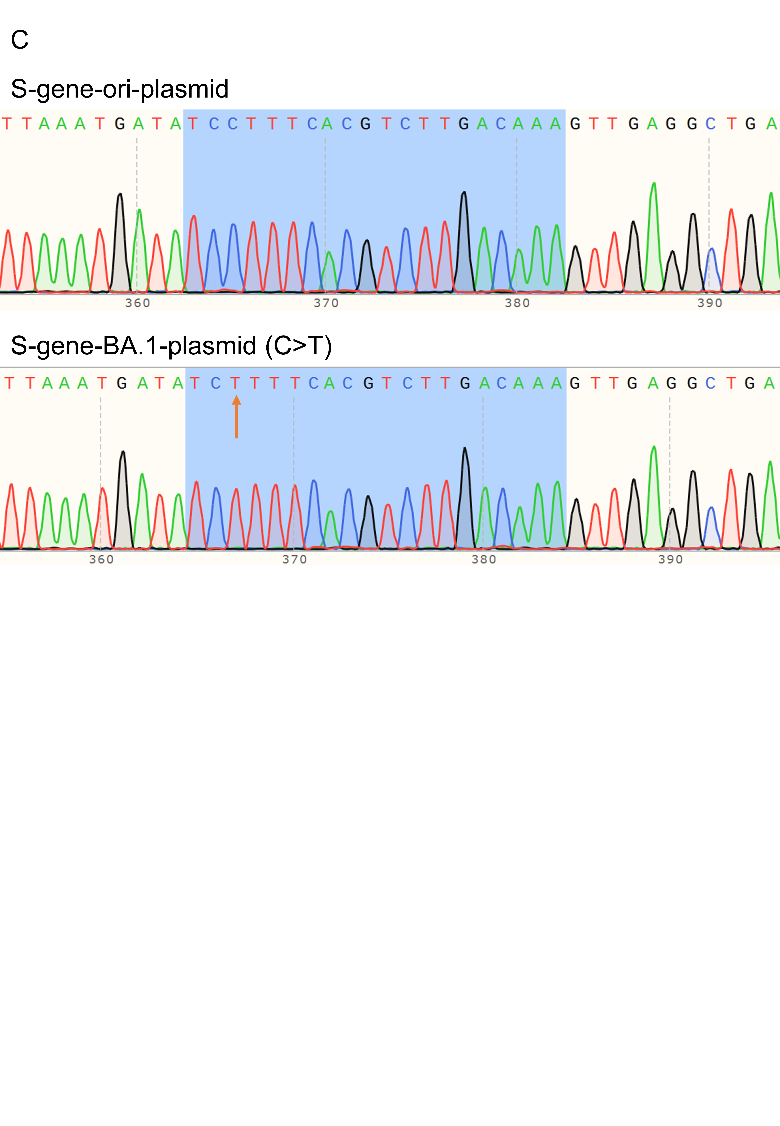


**Fig. S1 Verification of successful mutation sites constructed on the three plasmids containing mutated sequences.** A, mutation of D950N (24410 G˃A) on the delta variant is verified; B, mutation of N969K (24469 T ˃ A) on the omicron variant is verified; C, L981F (24503 C ˃ T) on the BA.1 variant is verified.


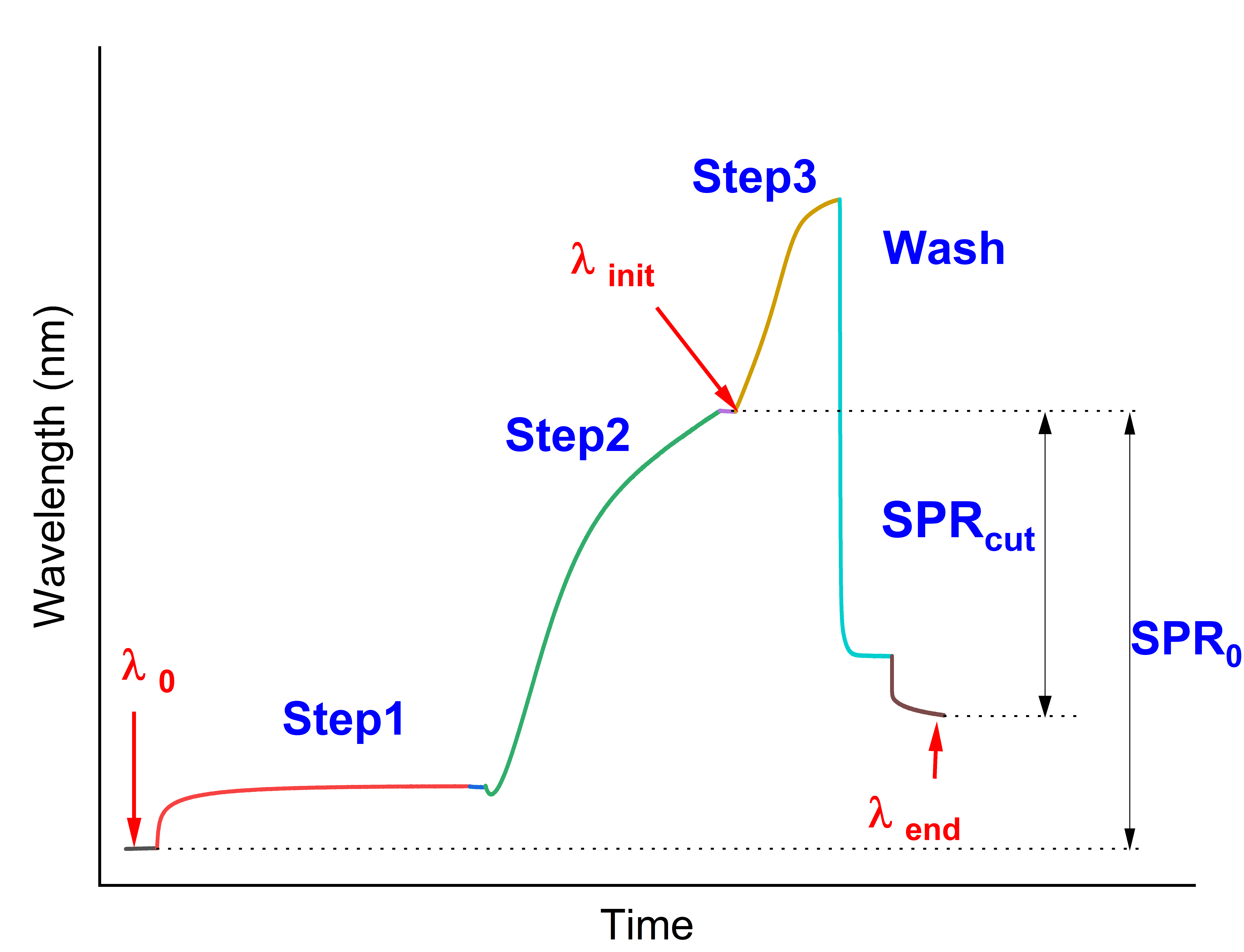


**Fig. S2 Detailed SPR signal during the experiment.** The relevant steps were stated in Results and Discussion- MOPCS on-device measurement.

**Reference**

1. Wang X, Zeng Y, Zhou J, Chen J, Miyan R, Zhang H, et al. Ultrafast Surface Plasmon Resonance Imaging Sensor via the High-Precision Four-Parameter-Based Spectral Curve Readjusting Method. Anal Chem. 2021;93(2):828–33.

2. Zhou J, Wang X, Chen J, Zeng Y, Gu D, Gao BZ, et al. Polymeric microsphere enhanced surface plasmon resonance imaging immunosensor for occult blood monitoring. Sensors Actuators B Chem [Internet]. 2022;350(July 2021):130858. Available from: https://doi.org/10.1016/j.snb.2021.130858

3. Broughton JP, Deng X, Yu G, Fasching CL, Servellita V, Singh J, et al. CRISPR–Cas12-based detection of SARS-CoV-2. Nat Biotechnol [Internet]. 2020;38(7):870–4. Available from: http://dx.doi.org/10.1038/s41587-020-0513-4

4. Hasan R, Hossain ME, Miah M, Hasan MM, Rahman M, Rahman MZ. Identification of Novel Mutations in the N Gene of SARS-CoV-2 That Adversely Affect the Detection of the Virus by Reverse Transcription-Quantitative PCR. Microbiol Spectr. 2021;9(1):4–6.

5. Kellner MJ, Koob JG, Gootenberg JS, Abudayyeh OO, Zhang F. SHERLOCK: nucleic acid detection with CRISPR nucleases. Nat Protoc [Internet]. 2019 Oct 23;14(10):2986–3012. Available from: http://www.nature.com/articles/s41596-019-0210-2
